# Correlation of Parathyroid Hormone Values With Lung Function Parameters in Patients With Chronic Obstructive Pulmonary Disease

**DOI:** 10.7759/cureus.64053

**Published:** 2024-07-08

**Authors:** Tanja Zovko, Kristina Galic, Marina Vasilj, Nikolina Pravdic, Ivanka Mikulic, Vinka Mikulic, Ante Mandic, Stanko Zovko, Danijel Pravdic

**Affiliations:** 1 Pulmonary Diseases and Tuberculosis, University Clinical Hospital Mostar, Mostar, BIH; 2 Neurology, University Clinical Hospital Mostar, Mostar, BIH; 3 Laboratory Diagnostics, University Clinical Hospital Mostar, Mostar, BIH; 4 Internal Diseases, University Clinical Hospital Mostar, Mostar, BIH; 5 Surgery, University Clinical Hospital Mostar, Mostar, BIH

**Keywords:** forced expiratory volume, • the maximal expiratory flow at 50% of the forced vital capacity, forced vital capacity, pulmonary function, vitamin d, parathyroid hormone, copd

## Abstract

Objectives: The aim of this study was to determine the disturbances in the concentration of parathyroid hormone (PTH) and 25-hydroxyvitamin D (vitamin D) in patients with stable chronic obstructive pulmonary disease (COPD) and its correlation with airflow obstruction.

Materials and methods: A prospective study included 200 patients with a confirmed diagnosis of COPD in the Department of Lung Diseases and Tuberculosis and Pulmonology Polyclinic of University Clinical Hospital Mostar in the period of three years, between May 2021 and May 2024. Inclusion criteria were a stable phase of COPD, hemodynamically stable patients older than 40 years, forced vital capacities in the first second (FEV1)/forced vital capacities (FVC) <0.7, and patients with PTH, vitamin D, calcium, and phosphate measurements. Exclusion criteria were acute exacerbation of COPD in the last month; current treatment with nutritional supplements, vitamins, and statins; lack of availability of lung function data; use of systemic corticosteroids in the previous three months; chronic renal insufficiency, respiratory diseases other than COPD (asthma, pneumonia, tuberculosis, and bronchiectasis), and other diseases (cancer and parathyroid disease). Medical records about demographic data (age and gender), pulmonary function test (FVC, FEV1, FEV1%FVC, mean expiratory flow (MEF)_50_), body mass index (BMI), COPD assessment test (CAT), Modified Medical Research Council (mMRC) Dyspnea Scale, and serum PTH, vitamin D, calcium, and phosphate levels were obtained.

Results: Patients with higher COPD stage had lower spirometry values, most significantly MEF_50_. The higher the COPD group (Global Initiative for Chronic Obstructive Lung Disease (GOLD) D), the lower vitamin D ​​and the higher PTH levels were. Calcium and phosphate values ​​were the same for all groups. Vitamin D and PTH levels significantly ​​correlated with MEF_50_ values. The lower MEF_50_ level, the higher PTH levels, ​​and lower vitamin D levels were found (P<0.05).

Conclusion: Our study showed that the patients in the higher COPD group have lower vitamin D levels ​​and higher PTH levels, indicating that they developed secondary hyperparathyroidism. The levels of vitamin D and PTH correlated the most with MEF_50 _values while other spirometry parameters did not significantly correlate with vitamin D and PTH levels.

## Introduction

Chronic obstructive pulmonary disease (COPD) is the third leading cause of death worldwide and the main incurable global health problem [1,2]. It is caused by chronic exposure to smoking and/or occupational or environmental factors [3,4]. The main characteristics of COPD are chronic bronchitis, chronic airway obstruction, and emphysema. The progression of the disease is connected to structural changes, airflow limitation, dynamic hyperinflation, peribronchial fibrotic remodeling of the lungs, and significant systemic inflammatory components [5]. COPD is divided into four groups (A, B, C, D) by the Global Initiative for Chronic Obstructive Lung Disease (GOLD) classification based on spirometry, modified dyspnea assessment scale - Medical Research Council Scale (mMRC) and COPD assessment questionnaire - COPD Assessment Test (CAT), and exacerbations [6]. It is not just a local lung disease but a systemic disease also [7].

Epidemiological studies confirmed the existence of many extrapulmonary comorbidities [8]. To understand and predict the complexity of the pathophysiology of the disease, efforts are made to identify biomarkers that correlate with disease severity. One of the proposed biomarkers is the concentration of serum 25-hydroxyvitamin D (vitamin D) [9].

Its levels correlate with food intake and regulatory mechanisms, such as parathyroid hormone (PTH). Vitamin D deficiency (VDD) is associated with a greater risk of chronic diseases like cancer, autoimmune diseases, cardiovascular diseases, and respiratory diseases [9]. The previous studies show inconsistent results regarding the role of vitamin D in pulmonary function [10]. It has an important role in the balance between calcium and phosphate, immunomodulation, anti-inflammatory action, and protection against infection. Deficiency is relatively common and is associated with frequent worsening and reduction of pulmonary function [11].

VDD has also been associated with decreased pulmonary function, increased inflammation, and decreased immunity. COPD poses a high risk for VDD, which is thought to be caused by malnutrition, insufficient outdoor activity, kidney dysfunction, and high catabolism associated with steroid therapy. On the other hand, VDD is also supposed to adversely affect pulmonary functions because, for optimal lung function, vitamin D seems required, beginning from the developmental stages. COPD patients are in a vicious circle of worse lung function due to VDD and decreased vitamin D levels due to COPD [12]. A low serum vitamin D leads to a decrease in calcitriol, which limits intestinal absorption of calcium generating a tendency toward hypocalcemia. This lowering of serum calcium increases PTH secretion, which stimulates the production of calcitriol in the kidneys, so that serum calcitriol is kept at normal levels at the expense of higher serum PTH (secondary hyperparathyroidism) [13]. Studies have shown that as a response to low levels of vitamin D, serum PTH is increased in some but not in all COPD patients [14]. So far according to our knowledge, there is an insufficient amount of data about the correlation between PTH and vitamin D changes in COPD with different parameters of lung function and in different stages of COPD. Therefore, the objective of this study was to correlate forced vital capacities (FVC), forced vital capacities in the first second (FEV1), FEV%FVC, and mean expiratory flow (MEF)_50_ values with PTH, vitamin D, calcium, and phosphate levels in patients with COPD groups from A to D.

## Materials and methods

Study design and study population

The prospective study included patients diagnosed with COPD who were treated in the Department of Lung Diseases and Tuberculosis and the Pulmonology Polyclinic of the University Clinical Hospital Mostar. The research was conducted over a period of three years, from May 2021 to May 2024.

Data collection

A total of 200 subjects diagnosed with COPD, aged between 40 and 80, were included in the research. According to the GOLD classification from 2021. they were divided into four groups: A) low-risk with fewer symptoms; according to GOLD: 1-2 spirometry results; number of exacerbations/year: ≤1 without indication for hospitalization; mMRC questionnaire: 0-1; CAT question paper: <10. B) Low-risk with more symptoms; according to GOLD: 1-2 spirometry results; number of exacerbations/year: ≤1 without indication for hospitalization; mMRC questionnaire ≥2; CAT questionnaire: ≥10. C) High risk with fewer symptoms; according to GOLD: 3-4 spirometry results; number of exacerbations/year: ≥2 or ≥1 with an indication for hospitalization; mMRC questionnaire: 0-1; CAT question paper: <10. D) High risk with multiple symptoms; according to GOLD: 3-4 spirometry results; number of exacerbations/year: ≥2 or ≥1 with an indication for hospitalization; mMRC questionnaire: ≥2; CAT questionnaire: ≥10.

Inclusion criteria were a stable phase of COPD, hemodynamically stable patients older than 40 years, FEV1/FVC <0.7, and patients with vitamin D, PTH, total calcium, and phosphates measurements. Exclusion criteria were acute exacerbation of COPD in the last month; current treatment with nutritional supplements, vitamins, and statins; lack of availability of lung function data; use of systemic corticosteroids in the previous three months; chronic renal insufficiency; respiratory diseases other than COPD (asthma, pneumonia, tuberculosis, and bronchiectasis); and other diseases (cancer and parathyroid disease).

Several variables were monitored and compared throughout the study. During the patient visits, anamnestic data was taken, and a physical examination was performed with a special focus on the examination of the respiratory system. Symptoms such as cough, shortness of breath and chest pain, smoking habits, and medication use were recorded. The subjects were classified as current and ex-patients who had never smoked. Body mass index (BMI) was calculated as body weight divided by the square of height (kg/m^2^). Demographic and clinical questionnaires contained patient gender, age, medical records, duration of COPD, frequency of exacerbations, and comorbidities. The clinical parameters that were monitored are the number of COPD exacerbations, spirogram, Medical Research Council (mMRC) Scale, six-minute walk test, and CAT [15].

Lung function was measured by spirometry. The following parameters were monitored: volume, time, and airflow. The patient was asked to inhale as deeply as possible, and then exhale into the sensor for as long as possible, preferably at least six seconds. Three technically appropriate measurements were made, whereby the variability between the two largest FVC and FEV1 must not exceed 5%. Spirometric testing was performed on a spirometer and Jaeger MasterScreen Body (Jaeger Corporation, Omaha, USA) [15,16].

The CAT questionnaire is a short and simple questionnaire that objectively measures the patient's quality of life. It was developed to ensure that questionnaire details measure the impact of COPD including cough, expectoration, activity limitation, sleep, fatigue, and psychological status. The results for individual items in the questionnaire provide insight into the impact of individual components of COPD on the quality of life and insight into problem areas [17,18].

The MRC Scale shortness of breath scale is graded from 1 to 4, where grade 0 is almost unrestricted (except for dyspnea in the highest loads), and grade 4 is the patient unable to leave the house due to lack of air even when dressing or feeding. Grade 1 is dyspnea when climbing the stairs to the fourth floor or up a hill, and grade 2 is classified as patients who are slower when walking on the level than their peers, while patients with grade 3 cannot walk more than 100 meters without stopping [19]. 

For the evaluation of airflow obstruction severity, the global initiative for chronic obstructive lung disease (GOLD) staging system, and the assessment of COPD, the “ABCD” combined assessment tool to the GOLD classification from 2021 was used. There were 50 patients in each group.

PTH was determined by standard laboratory methods at the Department of Laboratory Diagnostics, University Clinical Hospital Mostar. The sample was venous blood taken from the cubital vein. It was determined with a plasma-EDTA sample by the electrochemiluminescence method using a Roche Cobas e411 analyzer. Vitamin D was determined by standard laboratory methods by the electrochemiluminescence method using the Roche Cobas e411 analyzer. The sample was venous blood taken from the cubital vein. Calcium and phosphates were determined from the serum by a photometric UV test method using a Beckman Coulter AU 680 analyzer. The sample was venous blood taken from the cubital vein. Samples were stored at -20°C until processing.

This clinical study was approved by the Ethics Committee of the University Clinical Hospital Mostar where the study was conducted (No. 688/20). Our study was performed following the ethical standards of the Helsinki Declaration of 1975, as revised in 2000.

Statistical analysis

For this work, differences in the average values of individual parameters that describe the stage of COPD disease were tested with the ANOVA test for dependent samples. For this test, data matching with a normal distribution was tested. The post-hoc Bonferroni test was used to test differences between groups. Pearson correlation was used to test the correlation between the data. Statistical analyses were performed using GraphPad Prism for Windows (Boston, MA, USA). P<0.05 was considered statistically different.

## Results

This prospective study included 200 patients with confirmed diagnosis of COPD in the Department of Lung Diseases and Tuberculosis and Pulmonology Polyclinic of University Clinical Hospital Mostar. The research included the period from May 2021 to May 2024. The total number of patients was 200 patients with diagnosed COPD. According to the GOLD classification from 2019, they were divided into four groups each having 50 patients (GOLD A, B, C, D). The patient characteristics are shown in Table 1. In all groups, there was no significant difference in age. Out of all respondents, the number of male patients was 135 (67.5%), and there were 65 (32,5%) female patients (P<0.05). The male patients were dominant in each group, while the number of active smokers was highest in the GOLD 1 group (70%), and past smokers were dominant in the GOLD 4 group (67%). The duration of COPD was the longest in the GOLD 4 group, while most of the patients with BMI greater than 25 kg/m^2 ^were in the GOLD 1 group (P<0.05).

**Table 1:** Characteristics of the patients. Shown are means±standard deviations, unless differently stated. *P<0.05 COPD, chronic obstructive pulmonary disease; GOLD, global initiative for chronic obstructive lung disease; BMI, Body mass index

COPD	GOLD 1		GOLD 2		GOLD 3		GOLD 4		
Number of patients	50		50		50		50		
Male/female	33/17		29/21		34/16		29/21		
Age (years)	65±7		67±7		67±8		69±8		
Smoking status (% of patients)	Current smoker	Past smoker	Current smoker	Past smoker	Current smoker	Past smoker	Current smoker		Past smoker
	70^*^	30	52	48	52	48	33	67	
Smoking (pack/year)	55±28		58±35		60±36		56±32		
The average duration of COPD (years)	4.0		4.9		6.3		6.6		
BMI >25 kg/m^2^ (% of patients)	66^*^		62		46		50		

**Figure 1:**
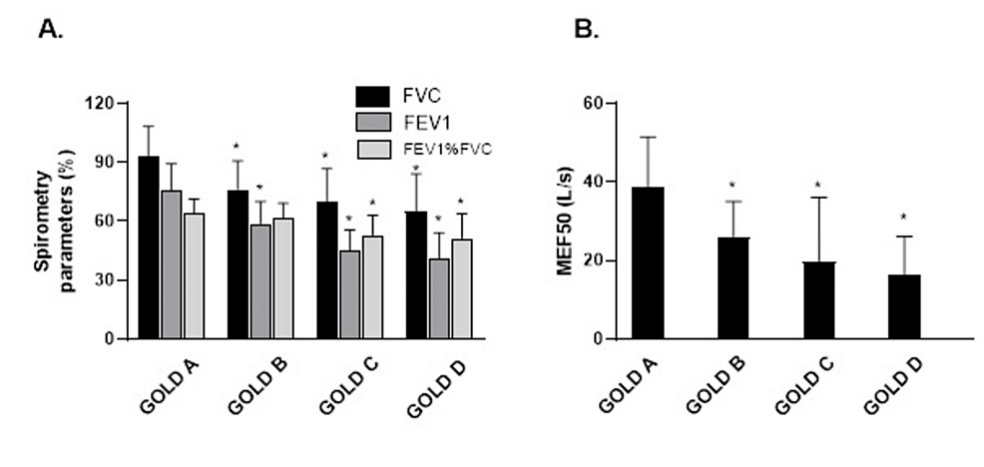
The mean values of FVC, FEV1, FEV1%FVC, and MEF50 depending on the GOLD grade. *P<0.05 FVC, forced vital capacity; FEV1, forced expiratory flow in the first second; MEF, mean expiratory flow; GOLD, global initiative for chronic obstructive lung disease

There was a significant difference in FVC, FEV1, FEV%FVC, and MEF_50_ in all groups when compared to the control group. Only in GOLD B there was no significant difference in FEV%FVC compared to GOLD A (Figure 1).

**Figure 2:**
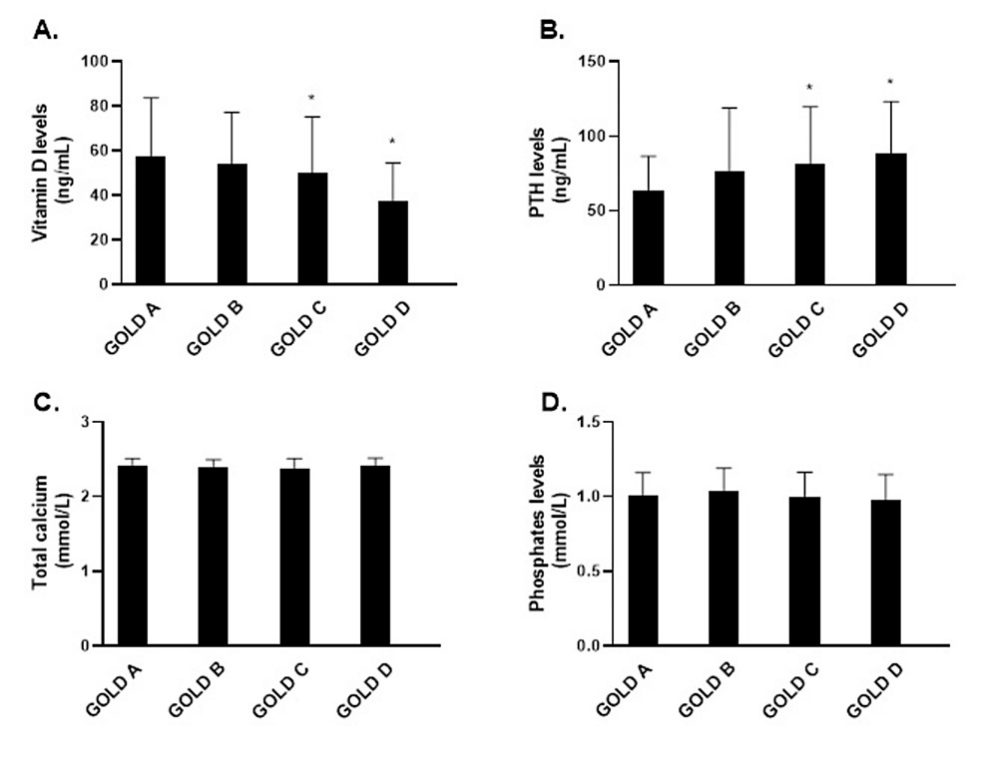
The mean values of vitamin D (A), PTH (B), total calcium (C), and phosphates (D) depending on the GOLD grade. *P<0.05 PTH, parathyroid hormone; GOLD, global initiative for chronic obstructive lung disease

There was a significant difference between vitamin D and PTH levels between GOLD A and GOLD C and GOLD D groups, respectively (Figure 2A and Figure 2B). The values of total calcium and phosphates were not different between the GOLD groups (Figure 2C and Figure 2D).

**Figure 3:**
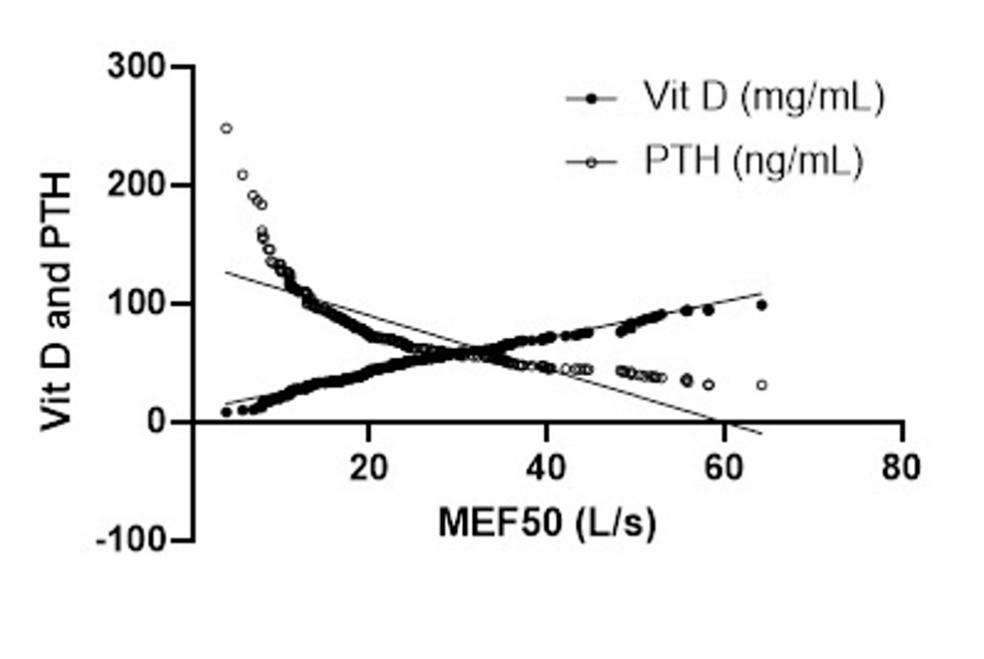
The correlation between vitamin D, PTH, and the maximal expiratory flow at 50% of the FVC (MEF50) levels. PTH, parathyroid hormone; FVC, forced vital capacity; MEF, mean expiratory flow

There was a significant correlation between the values of MEF_50_ and vitamin D and PTH levels (P<0.05). The patients with lower values of MEF_50_ had significantly lower levels of vitamin D and significantly higher levels of PTH. When other parameters (FVC, FEV1, and FEV1%FVC) were tested, there was a positive correlation between MEF_50_ and FEV1 value but it did not reach the significant level (P<0.08) (Figure 3).

## Discussion

In this prospective study of 200 subjects, we analyzed the association between levels of serum vitamin D, PTH, calcium, phosphate, and COPD severity. The COPD is a systemic and not just a local pulmonary disease [8]. Epidemiological studies confirm the existence of various extrapulmonary comorbidities such as metabolic disorders. The number of patients who die due to the consequences of systemic comorbidities is increasing, more so than from respiratory failure, and metabolic disorders are common extrapulmonary comorbidities [8,20-22]. So far, great efforts have been made to identify biomarkers that correlate with disease severity, and one was the concentration of serum vitamin D. Its level is affected by food intake and regulatory mechanisms, such as PTH. The association between vitamin D and COPD is not well studied and has yet to be fully investigated [23,24]. Studies show inconsistent results regarding the role of vitamin D in pulmonary function [10]. Its deficiency is relatively common and is associated with frequent worsening and reduction of pulmonary function [11]. VDD is associated with risks of chronic diseases like cancer, autoimmune diseases, cardiovascular diseases, and respiratory diseases [24]. The studies carried out so far suggest that VDD may be a risk factor for respiratory disease and that lower levels of vitamin D are related to reduced levels of pulmonary function measured by FEV1 and FVC [23]. VDD is highly prevalent, but the relation between serum vitamin D levels, and COPD severity is not yet well documented [25]. Also, to our knowledge, there were a few studies that evaluated the relationship between PTH, pulmonary function, and COPD [26]. One study reported prevalence of VDD in COPD patients was 89.3%, and secondary hyperparathyroidism associated with VDD was 22.9% [14]. The study done by Park JH and coauthors investigated that PTH values independently correlated with decreased FVC (% predicted) and FEV1 (% predicted) and that vitamin D levels were neither associated with pulmonary function [10]. The results of our study showed that patients in the higher COPD group have lower vitamin D and higher PTH levels. The levels of vitamin D and PTH levels correlated the most with MEF_50_ values. Our results support the thesis that PTH is independently associated with pulmonary function in COPD. In previous studies, vitamin D was regarded as a possible biomarker for COPD. Many recent studies produced controversial results. Until now, studies focused on the role of vitamin D without realizing the impact of PTH in these patients and PTH could be a better biomarker, especially in parts where VDD is in high prevalence [10]. Different complications can develop as a consequence of secondary hyperparathyroidism including osteoporosis, parathyroid hyperplasia, tertiary hyperparathyroidism, immune dysfunction, and different cardiovascular complications. All these effects could further worsen the existing lung disease. So, monitoring of vitamin D levels should be performed in COPD patients and, if necessary, the vitamin D values corrected [27]. The limitation of our study is that it is a single-center study and has a relatively small number of patients. So the results must be investigated in multicentric studies with a higher number of patients coming from different countries and ethnic groups.

## Conclusions

The patients in the higher COPD group have lower vitamin D and higher PTH levels. The levels of vitamin D and PTH levels correlated the most with MEF_50_ values. Our results support the thesis that PTH is independently associated with pulmonary function in COPD patients. Future studies are needed to explore this relationship in more detail. The reliable biomarkers that can be used as predictors of COPD outcomes should be identified.
